# Prevalence and prognosis of acutely ill patients with organ failure at arrival to hospital: protocol for a systematic review

**DOI:** 10.1186/s13643-017-0622-4

**Published:** 2017-11-15

**Authors:** Peter Bank Pedersen, Asbjorn Hrobjartsson, Daniel Lykke Nielsen, Daniel Pilsgaard Henriksen, Mikkel Brabrand, Annmarie Touborg Lassen

**Affiliations:** 1 Department of Emergency Medicine, Institute of Clinical Research, University of Southern Denmark and Odense University Hospital, DK-5000 Odense C, Denmark; 2 Centre for Evidence-Based Medicine, University of Southern Denmark and Odense University Hospital, DK-5000 Odense C, Denmark; 3 Department of Emergency Medicine, Odense University Hospital, DK-5000 Odense C, Denmark; 4 Department of Emergency Medicine and Department of Respiratory Medicine, Odense University Hospital, DK-5000 Odense C, Denmark; 5 Department of Emergency Medicine, Odense University Hospital, DK-5000 Odense C, Denmark; 6 Hospital of South West Jutland, DK-6700 Esbjerg, Denmark

**Keywords:** Organ failure, Emergency department, Emergency medicine, Acute medicine, Arrival, Prevalence, Prognosis

## Abstract

**Background:**

Acutely ill patients are a heterogeneous group, and some of these suffer from organ failure. As the prognosis of organ failure improves with early treatment, it is important to identify these patients as early as possible. Most studies on organ failure have been performed in intensive care settings, or on selected groups of patients, where a high prevalence and mortality have been reported. Before patients arrive to the intensive care unit, or the general ward, most of them have passed through the emergency department (ED), where diagnosis and treatment has been initiated. The prevalence and prognosis of acutely ill patients, with organ failure, at arrival have been studied in some selected groups, but methods and results differ. This systematic review aims to identify, summarize, and analyze studies of prevalence and prognosis of new onset organ failure in acutely ill undifferentiated patients, at arrival to hospital. The result of the review will assist physicians working in an ED, when assessing patients’ risk of organ failure and their associated prognosis.

**Methods:**

The information sources used are electronic databases, PubMed, Cochrane Library, EMBASE, and CINAHL; references in included studies and review articles; and authors’ personal files. One author will perform the title and abstract screening and exclude obviously ineligible studies. By an independent full-text screening, two authors will decide on the eligibility for the remaining studies. Eligible studies will include an unselected group of acutely ill adult patients at arrival to hospital, with one or more organ failures (respiratory, renal, cerebral, circulatory, hepatic, or coagulation failure). Included studies will have assessed the prevalence or prognosis, defined as mortality or ICU transfer, of new onset organ failure. From included studies, bibliographical and study description data, patient characteristics, and data related to prevalence of organ failure and prognosis will be extracted. We will assess risk of bias in included studies using the Quality in Prognosis Studies tool for prognostic studies and the Newcastle-Ottawa Scale for observational studies. We expect heterogeneity and to conduct a qualitative synthesis of the results. If, however, heterogeneity is low, we will conduct a random effects meta-analysis stratified by basic study design.

**Discussion:**

This review will summarize and analyze studies of prevalence and prognosis of acutely ill patients, with organ failure at arrival to hospital, assist ED physicians assessing the risk of organ failure in unselected patients, and guide recommendations for further research.

**Systematic review registration:**

PROSPERO CRD42017060871

**Electronic supplementary material:**

The online version of this article (10.1186/s13643-017-0622-4) contains supplementary material, which is available to authorized users.

## Background

Patients arriving with an acute condition are a heterogeneous group, and the initial step is to identify the critically ill. Some patients have organ failures based on different etiologies, affecting different organs with diverse severity, and identifying these is also a priority. Most of the existing knowledge regarding organ failure has been obtained in intensive care settings or on selected groups of patients, not on undifferentiated patients at the hospital doorstep, but later on.

Studies on patients in intensive care units (ICU) have reported a high prevalence of at least one organ failure (51–72%) at some point during the ICU stay. Respiratory organ failure is most prevalent, affecting as many as 87% organ failure patients. Prognosis described as in-hospital, short-term, and long-term mortality depends on the severity of organ dysfunction, number of failing organs, and the specific organ affected, and 5-year mortality is described at approximately 60% [1–4].

Outside the ICU, prevalence of organ failure, in unselected ward patients or after injury in US trauma and non-trauma centers, is described at approximately 7–14%. In undifferentiated patients with shock, the presence of organ failures has been described as 70%, at arrival to the emergency department. Organ failure is associated with increased in-hospital mortality, which increases with each additional organ failure, ranging from 12 to 60% with one to more than three organ failures [5–7].

Organ failure or the number of organ failures is also a risk factor in patients with severe sepsis [8]. There has been demonstrated an association between increasing number of organ failures and increased in-hospital, short-term, and long-term mortality and an association with ICU transfer from the emergency department. Likewise, it has been demonstrated that presence of clinically recognizable signs of organ failure results in better treatment compared to organ-specific laboratory values. The most common organ failures have been described as cardiovascular, renal, and respiratory [9–13]. Sepsis survivors have showed rates of persistent and long-term (≥ 90 days) organ dysfunction to be no less than 27 and 21% respectively [14].

Patients suffering from organ failure have to be identified and treated as early as possible. Before patients arrive to the ICU or general ward, most have passed the emergency department (ED) where the ED doctors have treated or started treatment due to acute illness. Furthermore, identifying patients at greatest risk for chronic organ failure is important, because it will allow early identification of susceptible patients which needs preventive interventions [14]. By this systematic review, we wish to identify, summarize, and analyze studies on the prevalence and prognosis of new onset organ failure in acutely ill undifferentiated patients at arrival on the hospital doorstep.

## Methods/design

We plan to conduct a systematic review. This protocol is reported according to the Preferred Reporting Items for Systematic Reviews and Meta-Analysis Protocols (PRISMA-P) 2015 statement (see checklist in Additional file 1) and developed with inspiration from the Cochrane Handbook for Systematic Reviews of Interventions [15–17].

### Objectives

Our overall aim is to assess the prevalence and prognosis of new organ failures in acutely ill patients at arrival to hospital. Our main research objective is to assess (1) the prevalence of new onset organ failures in acutely ill patients at arrival to hospital and (2) the prognosis of patients with newly onset organ failure at arrival to hospital.

### Eligibility criteria

#### Study designs

We will include observational studies (cross-sectional studies, prospective and retrospective cohort studies, and case-control studies), and randomized and non-randomized controlled trials, assessing the prevalence of new organ failure, or prognosis of acutely ill patients at hospital arrival. We will exclude case reports and studies with less than 100 patients.

#### Participants/population

Eligible studies will include an unselected group of acutely ill adult patients. Studies on selected groups of patients, such as specific conditions or diseases, will be excluded. Studies not restricted to adults are eligible and will be included in our analyses, provided that separate adult-data evaluation is achievable.

Studies on acutely ill patients, with one or more of the following organ failures, will be included: respiratory failure, renal failure, cerebral failure, circulatory failure, hepatic failure, or coagulation failure.

#### Outcomes

Studies that assess the prevalence of one or more organ failures, disregarding how prevalence has been defined or measured, will be included. Studies that have assessed prognosis, defined as mortality (short-term, long-term, and all-cause mortality and organ failure-specific mortality) or ICU transfer, will be included as well. Included studies may have dissimilar definitions of organ failures and may define short-term and long-term mortality by different thresholds. We will adhere to the organ failure definition as reported in the individual study and aim to extract mortality data as close as possible for 30-day and 1-year mortality.

#### Setting

We will include studies of patients which arrive to hospital at an emergency department, a trauma centre, a general ward, an acute medical unit, or other entrances for acutely ill patients. Studies where patients arrive directly at an intensive care unit are excluded.

#### Language

Studies published or conducted in English, or other languages the author group are able to read, will be included. Study titles in other languages will be listed and provided as an appendix if they seem relevant.

### Information sources

Information sources used are electronic databases not restricted to a specific period of time, references in included studies and review articles, and authors’ personal files. The databases we will use are PubMed, Cochrane Library, EMBASE, and CINAHL, and the protocol database, PROSPERO, will be searched for ongoing or recently completed systematic reviews on similar topics.

### Search strategy

Search strategy for the databases will be developed iteratively by the input from all the members of the project team and with the help from an information specialist from The Medical Research Library at the University of Southern Denmark in a face-to-face meeting.

We will systematically scan the reference lists of included studies or reviews for eligible studies and inspect authors’ personal files. We will not contact content experts for a list of possibly eligible studies.

When a search has been performed at one electronic database, the same search will be performed at the other databases and only adapt the exact subjects and syntax, which fit in that particular database. Just before finalizing the review, the search will be updated to ensure the most recent relevant studies are included. To increase transparency, a research record table (Table 1) will be included.

**Table 1:** Research record table

Information source	Date searched	Search	References	Comments
ᅟ	ᅟ	ᅟ	ᅟ	ᅟ

Draft PubMed:

- Organ failure
- Organ failure OR organ failures
- Organ failure OR organ failures OR organ dysfunction
- Organ failure OR organ failures OR organ dysfunction OR organ dysfunctions
- Organ failure OR organ failures OR organ dysfunction OR organ dysfunctions OR organ system dysfunction
- Organ failure OR organ failures OR organ dysfunction OR organ dysfunctions OR organ system dysfunction AND emergency department
- Organ failure OR organ failures OR organ dysfunction OR organ dysfunctions OR organ system dysfunction AND (emergency department OR emergency room)
- Organ failure OR organ failures OR organ dysfunction OR organ dysfunctions OR organ system dysfunction AND (emergency department OR emergency room OR acute medical unit)
- Organ failure OR organ failures OR organ dysfunction OR organ dysfunctions OR organ system dysfunction AND (emergency department OR emergency room OR acute medical unit OR non-ICU)

### Study records

#### Data management

References, from the literature search, will be exported to the software program “Endnote,” where a check for duplicates is performed. Afterwards, references are transferred to the software program “Covidence.org” for further processing. An internal study audit will be performed on studies conducted by identical authors to avoid double counting, and studies on identical data will be compared to clarify inconsistencies.

#### Selection process

PBP will perform title and abstract screening and exclude obviously ineligible studies. Other studies will be read in full length, independent, and in duplicate by two review authors, PBP and DLN. Subsequently, in agreement, the two review authors will decide whether the study meets inclusion criteria. The proportion of agreement is presented in the final review. Disagreements will be discussed at a face-to-face meeting, and in the case of continued disagreements, AH and ATL’s point of view will decide for the inclusion. Reasons for excluding full-text studies will be documented.

### Data collection process and data items

From included studies, we will extract bibliographical and study description data, patient characteristics, and data related to prevalence of organ failure and prognosis. Data extraction will be performed, independent and in duplicate by two reviewers, using predefined data fields. After pilot data extraction involving two to three studies, the data extraction sheet will be revised. In case of discrepancies, the rest of the review group is involved. In the absence of complete description of data or outcomes, we will attempt to establish contact to the authors by e-mail, as many as three times.

### Outcomes and prioritization

The primary outcome will be number of organ failures, and patients with organ failures at arrival (first recorded vital and laboratory values within 24 h or as close as possible) per 1000 visits. Organ failures studied are respiratory, circulatory, renal, hepatic, coagulatory, and cerebral, with any definition per original paper.

The secondary outcome, for patients with organ failure, is prognosis, assed by proportion and relative risk of transfer to ICU and/or 30-day (or as close as possible) and 1-year (or as close as possible) all-cause mortality.

### Risk of bias in individual studies

To assess the risk of bias within included studies, the Quality in Prognosis Studies (QUIPS) tool for prognostic studies, the Newcastle-Ottawa Scale (NOS) for observational studies, and the Cochrane Risk of Bias Tool (CRBT) for randomized controlled trials will be used. The QUIPS tool rates six bias domains: study participation, study attrition, prognostic factor measurement, outcome measurement, study confounding, and statistical analysis and reporting, as having high, moderate, or low risk of bias [18]. The NOS evaluate selection, comparability, and outcome in case-control and cohort studies by assigning stars [19]. The CRBT rates the following: selection bias, performance bias, detection bias, attrition bias, reporting bias, and other biases as low, high, or unclear [20]. Two independent reviewers, PBP and DLN, will assess every included study for bias; neither of the assessors is blinded to the studies, and disagreements will be resolved by discussion and eventually by consulting ATL and AH from the review group.

### Data synthesis

On basis of scoping searches conducted in preparation for this review, we do not anticipate to conduct a meta-analysis, as considerable diversity in studies to be included is expected. In any case, we will explore reasons for the expected heterogeneity according to clinical (participants and outcomes) and methodological (design and risk of bias) characteristics. Statistical test for heterogeneity will be performed (Cochran’s Q), and degree of heterogeneity will be described with the *I* ^2^ statistic. In the case of substantial and statistically significant heterogeneity (*I*-square > 60%, *P* < 0.10), a meta-analysis with the aim of assessing a weighted average will not be performed [21].

If heterogeneity is low, contrary to expectation, we will conduct a random effects meta-analysis, stratified by basic study design. We will present both synthetic and analytic views and try to explain heterogeneity by study characteristics and population characteristics.

We anticipate to perform a qualitative synthesis of the included studies (reporting median and interquartile results) based on tables and graphs to sum up results and findings. The synthesis will include basic study characteristics, results (including heterogeneity), risk of bias assessments, and explorations of reasons for heterogeneity [22].

In subgroups of patients with organ failure following trauma, bleeding, cardiac failure, or sepsis, sub-group analysis will be performed based on patient characteristics, types, and numbers of organ failure.

### Confidence in cumulative evidence

We will assess certainty of evidence provided by our review, inspired by the Grading of Recommendations Assessment (GRADE approach) depending on the basic design of the included studies and any down- or upgrading decisions as high, moderate, low, or very low. We will use the following assessment criteria when considering downgrading the certainty of the evidence: risk of bias, inconsistency, indirectness, imprecision, and publication bias to decide how to grade the certainty of evidence [23, 24]. As our study is observational by nature and do not address effect, we will not upgrade evidence based on standard criteria. The assessments will be performed by two review authors, PBP and DLN, independently, and disagreements will be resolved by discussion.

### Amendments

In case of protocol amendments, a table will be added with a description of every change, the rationale, and a date. Changes will not be incorporated into the protocol. Significant amendments will be registered in the PROSPERO register, and approval by all authors is required prior to registration. The review will contain a section describing differences between protocol and review.

## Discussion

The overarching goal of this review is to summarize data on prevalence and prognosis for acutely ill patients with organ failure at arrival to hospital. As early identification and treatment of these patients improves prognosis, systematic knowledge of the epidemiology might help clinical treatment in the emergency department. This has the potential to change the direct clinical management, as well as provide information of where to increase resources for the immediate management of acutely ill patients with organ failure.

This systematic review is planned to be published in a peer-reviewed journal.

## Additional file

Additional file 1: PRISMA-P 2015 checklist. (DOCX 35 kb)

## Abbreviations

CRBT: Cochrane Risk of Bias Tool
ED: Emergency department
GRADE: Grading of Recommendations Assessment
ICU: Intensive care unit
NOS: Newcastle-Ottawa Scale
PRISMA-P: Preferred Reporting Items for Systematic Reviews and Meta-Analysis Protocols
QUIPS: Quality in Prognosis Studies
